# Radiological Stage of Hemophilic Arthropathy of Knee Does Not Correlate With Clinical Variables

**DOI:** 10.1111/hae.70088

**Published:** 2025-07-23

**Authors:** Arman Vahabi, Erdem Er, Abdussamet Kuyucu, Elcil Kaya Biçer, Semih Aydoğdu

**Affiliations:** ^1^ Department of Orthopaedics and Traumatology Ege University School of Medicine Izmir Turkey; ^2^ Department of Orthopaedics and Traumatology EMOT Hospital Izmir Turkey

**Keywords:** arthroplasty, bleeding disorder, end‐stage arthropathy, hemophilia, total knee replacement

## Abstract

**Background:**

Various functional and radiological systems have been developed to evaluate joint health and the severity of arthropathy in hemophilic patients. Clinical relevance of these radiological systems has received minimal attention.

**Methods:**

Through a retrospective chart review, 84 patients with end‐stage hemophilic arthropathy of the knee were included. Pettersson and Arnold–Hilgartner scores were assessed by two observers. Correlation between radiological stage and range of motion (ROM), KSS function subscores, KSS pain subscores, total KSS, and surgical time were analyzed.

**Results:**

Interobserver correlation was found to be very strong for Pettersson score, with a value of 0.84 (95% CI: 0.75–0.89) and moderate for the Arnold–Hilgartner with kappa value of 0.4 (95% CI: 0.17–0.62). Correlation between Pettersson score and clinical variables revealed a very weak inverse correlation with ROM (*r* = −0.12, 95% CI: −0.33 to 0.10), KSS pain (*r* = −0.09, 95% CI: −0.31 to 0.12), and KSS function (*r* = −0.16, 95% CI: −0.36 to 0.06), and weak inverse correlation with KSS (*r* = −0.24, 95% CI: −0.44 to 0.2). Regarding the correlation between Arnold–Hilgartner score, a weak inverse correlation was observed with ROM (*r* = −0.21, 95% CI: −0.41 to 0.00), and very weak inverse correlations with KSS pain (*r* = −0.06, 95% CI: −0.16 to 0.27), KSS function (*r* = −0.00, 95% CI: −0.22 to 0.21), and KSS (*r* = −0.04, 95% CI: −0.25 to 0.18).

**Conclusion:**

Radiologic staging systems demonstrate weak correlation with clinically meaningful variables in patients with end‐stage hemophilic arthropathy of knee, an important consideration in the management of hemophilic arthropathy.

## 1 Introduction

Hemophilia is an X‐linked genetic disorder characterized by deficiencies in clotting factors, resulting in a tendency to bleeding. Repetitive intra‐articular hemorrhages contribute significantly to joint damage, which is major cause of morbidity in patients with hemophilia. Over the past few decades, advancements in effective factor replacement therapies have led to substantial improvements in joint health of individuals with hemophilia, particularly among younger generations [1]. However, cases of end‐stage joint damage continue to be observed, albeit to a limited extent, especially in settings where factor replacement therapy has not been consistently accessible, in patients who did not receive adequate treatment during childhood, and in those who have developed inhibitors to factors [2].

The pathogenesis of arthropathy in hemophilia is characterized by a vicious cycle of intra‐articular hemorrhage, synovitis, and recurrent hemorrhage [3]. Although molecular mechanisms of this process remain incompletely identified, the cumulative detrimental effects of repeated intra‐articular bleeding on joint are well established. In joints that are subject to hemophilic arthropathy, typical findings include soft tissue contractures, bone density loss, epiphyseal enlargement, and changes in patellar, trochlear and condylar anatomy [4, 5, 6, 7, 8]. These distinctive alterations in bone and soft tissue characteristics differentiate surgical interventions in hemophilic knees from those performed for primary osteoarthritis, necessitating special considerations in preoperative, intraoperative, and postoperative management [9, 10, 11].

In many fields of orthopedic practice, radiological scoring and classification systems are important tools for providing objective and reproducible data that support various applications, including treatment planning, establishing indications for surgical interventions, and academic reporting. In arthroplasty procedures, decision for surgery is typically based on a comprehensive synthesis of clinical assessments and radiological findings. Whereas reliability and validity of scoring systems and the correlation between functional scores/symptoms and radiological grading have been reported for primary knee osteoarthritis [12, 13, 14, 15, 16], the reliability of scoring systems and the relationship between functional scores/symptoms and radiological staging and their clinical relevance in knees affected by hemophilic arthropathy have been inadequately reported [17, 18, 19]. Although previous studies have demonstrated a moderate correlation between the Hemophilia Joint Health Score (HJHS) and Pettersson scores, the HJHS score holds limited value in guiding orthopedic surgical decision‐making and is primarily useful for the longitudinal monitoring of early‐stage joint pathology [20, 21]. We aimed to investigate whether current radiological staging systems for hemophilic arthropathy of the knee correlate with clinical variables such as range of motion (ROM), pain status, and knee‐specific function scores in patients with end‐stage arthropathy. Additionally, we explored the reliability of the Pettersson and Arnold–Hilgartner scoring systems for assessing end‐stage hemophilic arthropathy of the knee when evaluated by experienced observers. Finally, we examined whether the radiological stage of the hemophilic knee is associated with surgical difficulty, which was defined as length of surgery.

## 2 Patients and Methods

The study was diagnostic study on prospectively collected and retrospectively analyzed data from single subspeciality hemophilic musculoskeletal care unit in an academic hospital.

The study population included hemophilic patients who had undergone total knee replacement due to end‐stage hemophilic arthropathy. Through a retrospective chart review, period of January 2002 to January 2021 was screened. The inclusion criteria comprised patients who underwent total knee arthroplasty for end‐stage hemophilic arthropathy and had complete clinical and radiological datasets. Exclusion criteria included a history of previous periarticular osteotomy, surgical procedures other than total knee arthroplasty, incomplete clinical or radiological data, or a diagnosis of a bleeding disorder other than hemophilia. During this period, all orthopedic interventions for hemophilia cases within our subspecialty unit were performed by a single senior arthroplasty surgeon. In cases of end‐stage hemophilic arthritis, the decision to proceed with total knee replacement was based on a variety of factors, including patient age, functional capacity, prior surgeries, and comorbid chronic conditions. Objective functional scores incorporated into the preoperative evaluation included the Tegner score, Knee Society Score (KSS), and KSS pain and function subscores. Standard radiological assessments were conducted using standard two‐way standing knee radiographs and standing long‐leg radiographs.

The Pettersson scoring system, proposed by Pettersson et al. in 1980 [22], aimed to provide an objective classification of joint damage severity in hemophilia cases, applicable across different joints rather than being knee specific. This system assigns a score from 0 to 13 based on radiographic parameters, including osteoporosis, epiphyseal enlargement, irregularity of the subchondral surface, joint space narrowing, subchondral cyst formation, erosion of the joint margin, incongruence of articulating bone ends, and joint deformity (Table 1).

**TABLE 1:** Parameters considered in hemophilic arthropathy staging systems.

Pettersson		Arnold–Hilgartner	
Parameter	Score	Definition	Stage
Osteoporosis	Absent: 0	Normal Joint	0
	Present: 1	Soft tissue inflammation	1
Enlargement of epiphysis	Absent: 0	Osteopenia and epiphyseal enlargement	2
	Present: 1	Changes in bony structures: Patellar squaring Widened notch Cyst formation	3
Irregular subchondral surface	Absent: 0	Narrowing of joint space	4
	Partial: 1	Substantial disorganization of joint: Joint contractures Loss of joint space Extensive epiphyseal enlargement	5
	Total: 2
Joint space narrowing	Absent: 0
	>1 mm: 1
	<1 mm: 2
Subchondral cyst	Absent: 0
	1 cyst: 1
	>1 cyst: 2
Erosion of joint margins	Absent: 0
	Present: 1
Gross incongruence in articulating surfaces	Absent: 0
	Slight: 1
	Pronounced: 2
Joint deformity (defined as angulation, displacement, or both)	Absent: 0
	Slight: 1
	Pronounced: 2

The Arnold–Hilgartner staging system, introduced by Arnold and Hilgartner [23], classifies joint damage based on broader criteria. This system assigns a grade from 0 to 5, following a nonknee‐specific approach. In this classification, a normal joint is graded as stage 0; soft tissue inflammation as stage 1; epiphyseal enlargement and osteopenia as stage 2; bone edge irregularity as stage 3; joint space narrowing as stage 4; and substantial joint disorganization as stage 5 (Table 1).

The Pettersson and Arnold–Hilgartner scores were independently assessed by two surgeons with experience in the preoperative evaluation and management of hemophilic cases on latest preoperative radiographs. For each scoring system, agreed values were measured from two measurements taken by each observer, spaced 2 weeks apart. Agreement was determined by assigning a score in a third round of evaluation for cases where discrepancies were found between the two initial measurements, conducted blindly to the results of the first assessment. Mean values of agreed scores for each observer were taken into consideration for correlation analyses.

Intraclass correlation coefficients (ICCs) were determined to evaluate the consistency between the observers' measurements. To assess the clinical relevance of radiological staging systems in cases of end‐stage hemophilic arthropathy, preoperative ROM, preoperative KSS function subscores, preoperative KSS pain subscores, and total KSS scores were obtained through chart review [24, 25, 26]. Their correlation with the mean Arnold–Hilgartner scores and mean Pettersson scores was then analyzed. Passive ROM was assessed using a goniometer preoperatively by a junior orthopedic resident. This assessment was considered as indirect measure of soft tissue contractures/stiffness.The correlation of surgical time and radiological stage of arthropathy  (which was used as an indicator to assess surgical difficulty) was also analyzed. This duration was defined as the time from the application of the tourniquet, tightened immediately before the incision following surgical site preparation, to its release after the application of a Jones bandage, which was applied following closure of incision. Correlation of surgical time with the radiologic stages was then analyzed.

### 2.1 Statistical Analysis

Data analysis was conducted using SPSS Statistics version 26 and MedCalc. The suitability of continuous variables for normal distribution were assessed using graphical methods (Q–Q Plot, Detrended Q–Q Plot) and the Shapiro–Wilk test. Continuous variables are summarized with mean and standard deviation, and categorical variables with counts and percentages. Inter‐rater agreement was evaluated separately for each radiologic scoring system. For the Pettersson score, which is a continuous numeric scale, inter‐rater reliability was assessed using the ICC with a two‐way mixed‐effects model for consistency with average measures (ICC [3, k]), and 95% confidence intervals were reported. For the Arnold–Hilgartner score, which is an ordinal staging system, Cohen's weighted kappa statistic with quadratic weights was used. To assess the clinical relevance of the radiological scoring systems, nonparametric Spearman correlation coefficients (rho) were calculated between the mean radiologic scores (Pettersson and Arnold–Hilgartner) and preoperative clinical variables: ROM, KSS pain, KSS function, and total KSS score and duration of surgery. All correlation results are presented with 95% confidence intervals. The 95% confidence interval for Cohen's kappa was computed using the standard error method: 𝜅 ± 1.96 × SE κ±1.96×SE, based on the asymptotic standard error provided by SPSS. A significance level of *α* = 0.05 was accepted. Correlation strength was classified as follows: 0.00–0.19 very weak, 0.20–0.39 weak, 0.40–0.59 moderate, 0.60–0.79 strong, and 0.80–1.00 very strong [27]. A power analysis was performed for two‐tailed Pearson correlation analysis using a target correlation coefficient of 0.30, an alpha level of 0.05, and a desired statistical power of 0.80. The analysis indicated that a minimum sample size of 84 subjects was required to detect a medium effect size with adequate power. The final sample of 84 cases met this threshold, yielding an achieved power of 80.03%

## 3 Results

A total of 110 patients underwent surgical treatment for end‐stage hemophilic arthropathy during the specified period. After excluding 2 patients who had previously undergone around‐knee osteotomies, 7 patients under follow‐up for bleeding disorders other than haemophilia, 2 patients who had undergone knee arthrodesis, and 15 patients with incomplete clinical or radiological data, 84 patients with end‐stage hemophilic arthropathy of the knee who received total knee replacement were included in the final analysis.

All cases involved male patients. All 84 cases were graded 7 or higher according to the Pettersson score and 4 or higher according to the Arnold–Hilgartner. Of these, 47 cases (56%) underwent total knee replacement on left knee, whereas 37 cases (44%) had operated on right side. Among the patients, 71 (85%) had hemophilia A and 13 (15%) had hemophilia B. All surgeries were performed under general anesthesia. The medial parapatellar approach was used in 82 cases (98%) for arthrotomy, whereas the lateral approach was used in 1 case (1%), and a combined medial and lateral parapatellar approach was employed in 1 case (1%). A total of 20 chronic comorbid conditions were identified in 17 patients. These included chronic hepatitis C virus (HCV) infection in eight patients, hypertension in six, epilepsy in two, coronary artery disease in two, chronic hepatitis B virus (HBV) infection in one, and a history of cerebrovascular accident (CVA) with sequelae in one patient (Table 2).

**TABLE 2:** Descriptive characteristics.

	Median	Minimum	Maximum
Age (in years)	41	22	62
Factor level (%)	1	0.1	7.1
Follow‐up time (in months)	24	2	180
Hip‐knee angle (in degrees)	175	150	227
Range of motion (in degrees)	65	0	120
Duration of surgery (in minutes)	100	60	180
Pettersson score	11	6	13
Arnold‐Hilgartner score	5	4	5
KSS Total Score	37	0	91
KSS pain score	20	0	45
KSS function score	60	0	90

In the intraclass correlation analysis of the agreed measurements obtained by both observers for the Pettersson score, the interobserver correlation was found to be very strong, with a value of 0.84 (95% CI: 0.75–0.89). In the correlation analysis of the agreed scores obtained by both observers for the Arnold–Hilgartner scoring system, weak/moderate correlation was found with kappa value of 0.39 (95% CI: 0.17–0.62).

**FIGURE 1:**
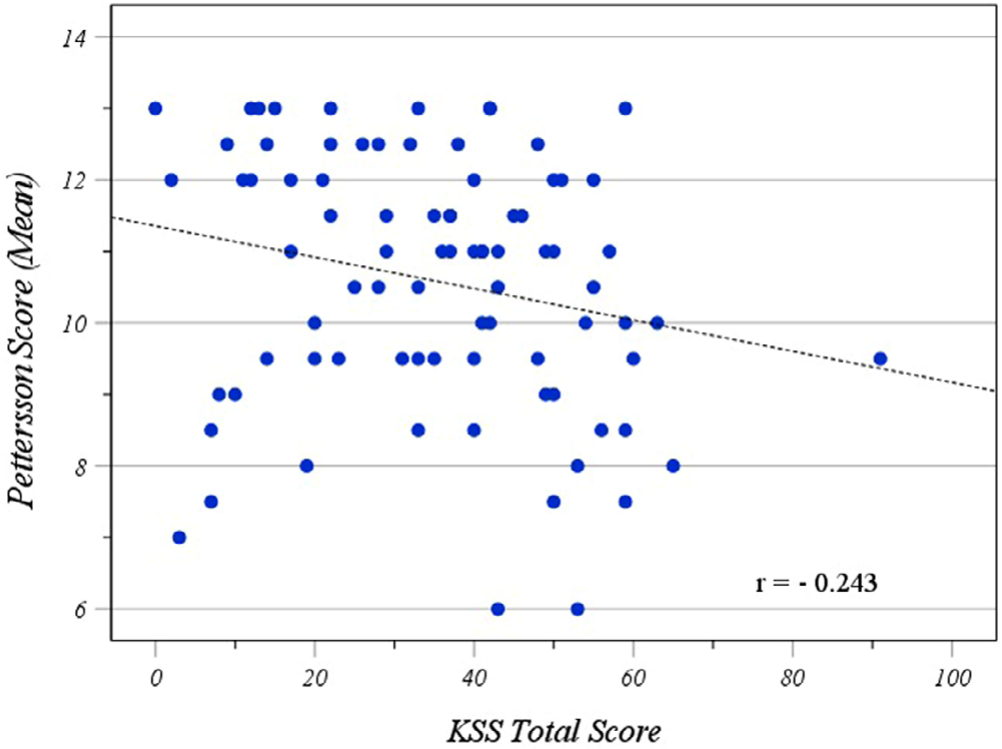
This diagram shows correlation of radiologic stage of arthropathy (according to Pettersson scoring system) with Knee Society Score (KSS Total Score).

Correlation between Pettersson score and clinical variables revealed a very weak inverse correlation with ROM (Spearman correlation coefficient [*r*] = −0.12, 95% CI −0.33 to 0.10), very weak inverse correlation with the KSS pain (*r* = −0.09, 95% CI: −0.31 to 0.12), a very weak inverse correlation with the KSS function (*r* = −0.16, 95% CI: −0.36 to 0.06), and weak inverse correlation with KSS (*r* = −0.24, 95% CI: −0.44 to 0.2). (Table 3) (Figure 1).

**TABLE 3:** Correlation analyses of staging of arthropathy with various functional scales and clinical variables.

			95% Confidence interval	
		Correlation coefficient	Lower bound	Upper bound
Pettersson score	Range of motion	−0.12	−0.33	0.10
	KSS pain subscale	−0.09	−0.31	0.12
	KSS function subscale	−0.16	−0.36	0.06
	KSS Total Score	−0.24	−0.44	0.02
Arnold–Hilgartner score	Range of motion	−0.21	−0.41	0.00
	KSS pain subscale	0.06	−0.16	0.27
	KSS function subscale	0.00	−0.22	0.21
	KSS Total Score	0.04	−0.25	0.18

*Note*: Correlation coefficients are based on Spearman's rho.

Abbreviation: CI, confidence interval.

Regarding the correlation between the mean Arnold–Hilgartner score and clinical variables, a weak inverse correlation was observed with ROM (*r* = −0.21, 95% CI: −0.41 to 0.00), along with very weak inverse correlations with the KSS pain subscale (*r* = −0.06, 95% CI: −0.16 to 0.27), KSS function subscale (*r* = −0.00, 95% CI: −0.22 to 0.21), and total KSS score (*r* = −0.04, 95% CI: −0.25 to 0.18) (Table 3).

**FIGURE 2:**
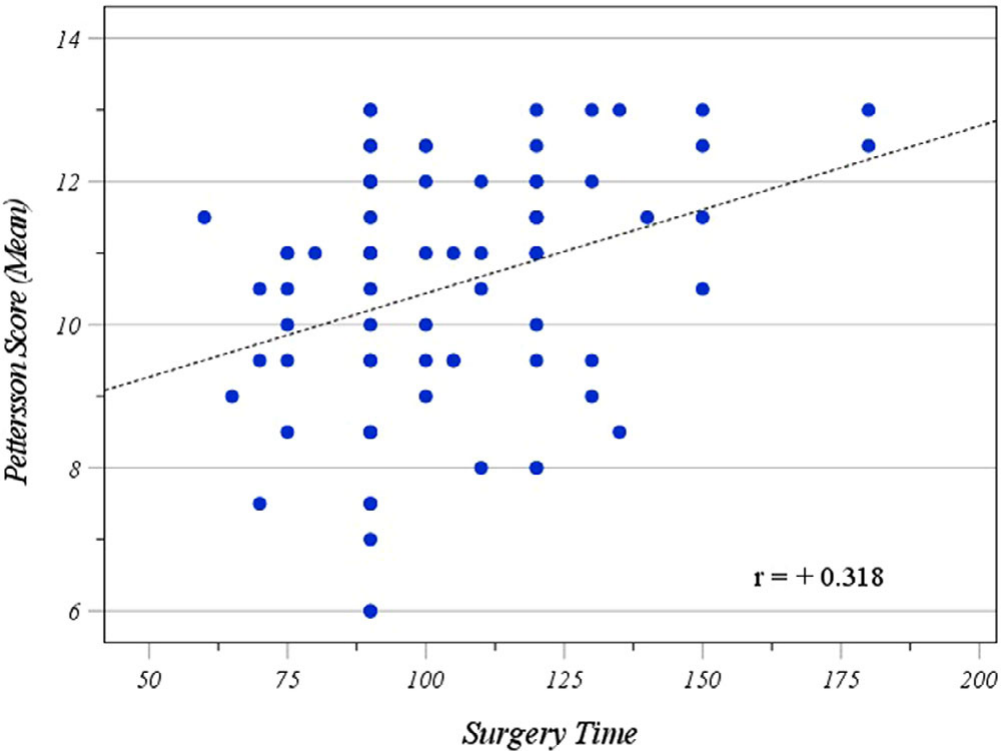
This diagram shows correlation of radiologic stage of arthropathy (according to Pettersson scoring system) with duration of surgery.

Surgical difficulty, as defined by surgical time, showed weak correlation with the Pettersson score (*r* = 0.31, 95% CI: 0.09–0.51) and very weak correlation with the Arnold–Hilgartner score (*r* = 0.08, 95% CI: −0.14 to 0.31) (Figure 2).

## 4 Discussion

The study demonstrated that in end‐stage hemophilic arthropathy of the knee, radiological stage shows only weak to very weak correlations with ROM and objective knee function assessment tools. Although there is no consensus on the correlation between pain and the radiological stage of arthritis in primary knee osteoarthritis, patients generally report activity‐related pain and secondary functional limitations [13, 15]. Pain during daily activities remains a significant factor in both patient complaint and the determination of surgical indications in such cases. However, in our study, the KSS pain subscores averaged only 20 out of 50 in end‐stage hemophilic cases, and the correlation between pain score and radiological stage was found to be very weak. This finding is consistent with our clinical observation that pain is neither as prominent nor as predictable in hemophilic arthropathy as it is in primary osteoarthritis. We propose that the low correlation between radiological and clinical variables in hemophilic arthropathy may not be solely due to limitations in existing staging tools but may instead reflect unique aspects of the disease's pathogenesis. This divergence suggests that radiological scoring in hemophilic arthropathy may not fully capture the clinical burden of the condition, an important consideration when planning surgery for these patients. Although studies in the literature have reported a moderate correlation between the HJHS and Pettersson scores, our study differs in context, as it focuses on patients with end‐stage hemophilic arthropathy, where the applicability of the HJHS score is limited [20, 21].

Our study demonstrated a very strong correlation between the agreed scores of two observers for both radiologic scoring systems. In the limited literature on this topic, there are diverse conclusions regarding the reliability of radiologic scoring systems for hemophilic joints, likely due to variations in study design and observer expertise [17, 19, 28, 29]. Specifically, factors such as nonjoint‐specific assessments, the inclusion of cohorts with a broader spectrum of joint status (encompassing both early‐ and end‐stage damage), and studies involving observers from different specialties should be considered before making definitive assessments on matter. Nevertheless, both our findings and the existing literature suggest that radiologic scoring in hemophilia achieves a reliable level of validity when conducted by experienced observers. We believe that certain scoring criteria, such as “irregular subchondral surface” in the Pettersson system and assessing osteoporosis from plain radiography, introduce an unavoidable element of subjectivity. Establishing expert consensus on a standardized atlas, or implementing minor modifications to established scoring systems, could help address these subjective factors and would be a valuable direction for future research [17].

In hemophilic knees, pathological changes include fibrotic fragility of periarticular soft tissues, which are typically resilient in healthy knees, and a decrease in bone density, resulting in softened bone that is normally denser in this age group [11, 30, 31, 32]. These alterations complicate intraoperative steps, such as achieving adequate exposure and ensuring soft tissue balance, in total knee replacement. In cases with more severe joint damage, advanced soft tissue contractures, and pronounced bone deformities–characteristics of more advanced disease stages–optimizing these intraoperative considerations would become increasingly challenging. However, the surgical time, which we used as an outcome measure for assessing surgical difficulty, showed only a weak correlation with the Pettersson score and a very weak correlation with the Arnold–Hilgartner score. We believe this may be because the primary challenges encountered during surgery are related to the fragility and stiffness of the periarticular soft tissue rather than the extent of joint damage itself. From this perspective, radiological scoring systems designed to assess bone, and joint damage may have limited value in predicting intraoperative challenges. Surgical difficulty has received limited attention in the field of orthopedics, whereas several studies from other surgical specialties have explored this concept, with some correlating it with operative time [33, 34, 35, 36]. We believe that assessing surgical difficulty warrants further discussion and investigation, especially in conditions with distinct pathogenesis, such as hemophilic arthropathy and rheumatoid arthritis.

The main limitation of this study was the selection bias arising from the exclusion of patients with early‐stage hemophilic arthropathy. This limitation restricted the evaluation of both scoring systems to end‐stage cases, framing all conclusions and discussions within the context of “end‐stage” hemophilic arthropathy. Another limitation was the measurement bias due to assessing surgical difficulty solely based on operative time. Although no established scale exists to quantify the surgical difficulty of knee replacement procedures, an analysis incorporating intraoperative and early postoperative complications might have provided a more comprehensive assessment. Another limitation of the study is its retrospective design spanning an extended period. However, we believe that this limitation was mitigated by the consistent use of standardized data collection forms and the continuous involvement of a single senior author throughout the study period. Additionally, potential confounders such as age, factor levels, and inhibitor status were not included in the correlation analysis. Nonetheless, as the study group consisted exclusively of patients with advanced hemophilic arthropathy due to more severe factor deficiency, we believe a reasonable level of clinical homogeneity was achieved, which helped reduce bias associated with these variables.

## 5 Conclusion

The structure of radiological staging systems for hemophilic arthropathy has been developed with the distinctive pathogenesis of this condition in mind. Although these tools are reliable when evaluated by experienced observers and might retain value for patient categorization and academic reporting, their clinical relevance is limited. These systems demonstrate a weak correlation with clinically meaningful variables, an important limitation to consider in the management of patients with hemophilic arthropathy of the knee.

## Author Contributions

Design: E.K.B. and S.A. Writing: A.V. and E.E. Data curation: A.K. and E.E. Review: E.K.B. and S.A.

## Ethics Statement

This study was conducted in accordance with Helsinki Declaration and its later amendments. Ethical approval for this study was obtained from Institutional Ethics Committee under code 24‐11T/56.

## Consent

Consent for participation and publication of the study was obtained from patients.

## Conflicts of Interest

The authors declare no conflicts of interest.

## Data Availability

Dataset for this study is available from corresponding author upon reasonable request.
